# Conceptual Design of Micro-Bioreactors and Organ-on-Chips for Studies of Cell Cultures

**DOI:** 10.3390/bioengineering5030056

**Published:** 2018-07-19

**Authors:** Carl-Fredrik Mandenius

**Affiliations:** Division of Biotechnology, IFM, Linköping University, 58183 Linköping, Sweden; cfm@ifm.liu.se; Tel.: +46-13-28-8967

**Keywords:** biomechatronic design, bioprocess development, toxicity testing, in vitro assay, drug testing, heart-on-a-chip, eye-on-a-chip

## Abstract

Engineering design of microbioreactors (MBRs) and organ-on-chip (OoC) devices can take advantage of established design science theory, in which systematic evaluation of functional concepts and user requirements are analyzed. This is commonly referred to as a conceptual design. This review article compares how common conceptual design principles are applicable to MBR and OoC devices. The complexity of this design, which is exemplified by MBRs for scaled-down cell cultures in bioprocess development and drug testing in OoCs for heart and eye, is discussed and compared with previous design solutions of MBRs and OoCs, from the perspective of how similarities in understanding design from functionality and user purpose perspectives can more efficiently be exploited. The review can serve as a guideline and help the future design of MBR and OoC devices for cell culture studies.

## 1. Introduction

The engineering design of micro-bioreactors (MBRs) and Organ-on-Chips (OoCs) has attracted much attention in recent years [1,2,3,4,5,6,7]. The two terms, MBR and OoC, have diverse origins. The MBR derives its name from the bioengineering methodology of performing biological reactions in micro-scale reactor devices; OoC refers to the recreation of organs and tissues from the human body on or in a miniaturized device with a smaller volume than the original organ and with body-like fluids streaming around the cells in an in vivo-like fashion [8,9,10]. Despite this difference, many of the basic engineering principles coincide in the design of MBRs and OoCs. Probably due to that, the terms are used concurrently.

The kind of problems addressed with MBRs and OoCs are related. For bioprocess development, the MBRs are considered valuable tools for accelerating development of new bioprocesses with microorganisms or mammalian cells as production organisms [11,12]. The culture of the manufacturing process is scaled-down to 1–10 mL volume of the MBR, and critical process parameters and media composition are systematically optimized [13,14]. The MBRs for bioprocess development have even been scaled down to the size of chips (Bioreactor-on-a-Chip) [15] and used for mimicking chemostat or turbidostat bioreactors with bacteria and yeast [15,16,17,18]. The increased yield and productivity of the large-scale process can be reached at a much earlier stage in the process development with this approach. Commercial MBRs with >100 parallel MBR units are now on the market, e.g., ambr [19] and m3p systems [20].

With OoC devices, the aim is to facilitate the study of organ cell assemblies in vitro, under conditions that recreate in vivo conditions of the organ in the body for recapitulating time-related cellular behavior. The OoC device allows for the observation of cellular effects when exposed to drugs or other chemicals. This allows for the assessment of compounds’ effects at subcellular and multicellular levels. Successfully applied, this supports the investigation of safety pharmacology and toxicology, and, when possible, the efficacy of drug compounds [7,8,9,10,21,22]. If OoCs generates reliable results, it will be important to control the cells’ survival in the artificial milieu of small-scale OoC; their stability relates to the design of liver devices [23,24,25]. However, other organs [26,27,28,29] have attracted almost the same interest, e.g., pancreas, eye, cartilage, heart, and lung cells [28,29], as well as combinations of several organ cell types on the same chip [30,31]. This is of great interest, as are tumors-on-chips, which are also covered in this special issue [32,33].

Importantly, the possibility to correctly observe, monitor, and analyze the effects of the cells through imaging, sensors, and other analytical means, as well as by controlling the process in the process development MBR or OoC, are pivotal for all applications of MBRs. Miniaturized sensors, optical fibers, and imaging inside or at the outlets of the MBRs/OoCs may provide these opportunities in a reliable way [34,35,36,37]. The design of the fluidics and transport inside the reactor chamber and the mixing in the device are common design problems. This results in some very similar design solutions.

**Figure 1:**
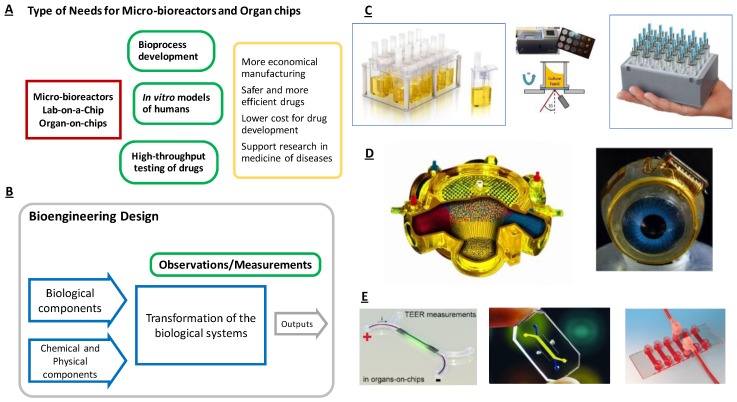
The similarities and diversities of micro-bioreactor design. (**A**) MBRs and OoCs have distinct purposes, which are expressed as user needs and requirements, e.g., the device should generate data for more economical manufacturing, safer and more efficient drugs, low cost for development, and support for medical research; (**B**) Despite the diversity in needs, a common transformation process can be outlined; (**C**) This is shared for MBRs used for process development; (**D**) for artificial liver bioreactors; and (**E**) for small plastic chips with multi-channels and internal membranes.

Figure 1 illuminates the similarities and diversities of MBR and OoC device for various applications. Although the intended use and outcome of the devices differ (Figure 1A), the transformations by the biological components in the devices are similar (Figure 1B). The common transformation process that occurs in every MBR device provides similar prerequisites for the design. The resulting design solutions, such as a rack of small MBR-containers with optical sensors placed at the bottom of each micro-vessel (Figure 1C); the compact artificial liver bioreactors with intertwined hollow-fibers for liquid and gas transport (Figure 1D); or small channels with an internal membrane for transepithelial electrical resistance measurement (TEER) for drug penetration studies, PDMS chips with double channels, and parallel channels (Figure 1E) are all examples that share the general structure in Figure 1B. Consequently, the design of the devices should follow that frame.

Thus, the engineering design of MBRs and OoCs have much in common, especially at a conceptual level. Established conceptual design methodology [38,39] could therefore significantly facilitate the development process of new MBR and OoC devices. The established conceptual design methodology is based on approaching the design of a new product from a functional perspective in which the functionality of the product drives the development of the design. In industrial design, conceptual methodology is widely applied to mechanical and electrical products [40]. However, in bioengineering it has been so far rarely used, with only a few examples on bioreactor scale-up [41,42], bioprocess configuration, monitoring and control [43,44], and stem cell production [45,46], but also recently for organ-on-chips [47].

In this review article, it is shown how the general conceptual design methodology can be applied to develop and improve the design of micro-bioreactors on a functional level. Two examples of conceptual design are shown to illuminate the similarities and differences in the design of MBR and OoC: (1) an MBR for production of hamster cells and (2) a heart-on-a-chip reactor for drug testing.

## 2. Conceptual Design Methodology

**Figure 2:**
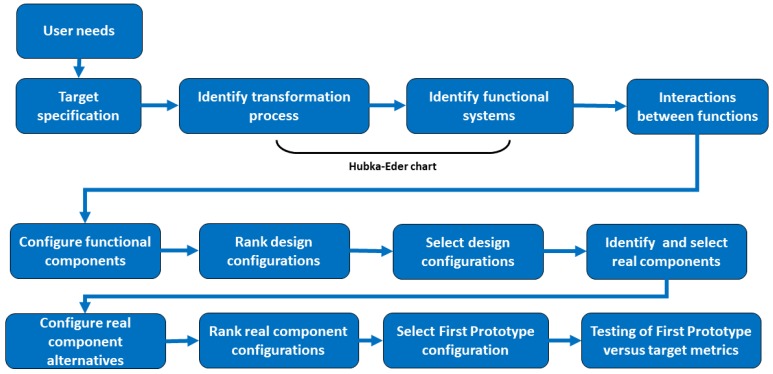
The workflow in conceptual design when developing a new product prototype as suggested by several authors [30,31,32]. The steps are iterative and partly parallel to speed up the work in the design team.

The conceptual design methodology is based on a systematic procedure to analyze the design objectives and, from these, conceive alternative design solutions that meet the objectives for a new product prototype [38,39,40,48]. The workflow in the development process in conceptual design starts with identifying the functions that are required to realize the user needs of the product and, from that, select and configure functional components that can effectuate user needs (Figure 2). Alternative configurations are compared with user needs and ranked versus user needs. This results in a preferred configuration. Once this choice is made, the functional components of the configuration are replaced with real physical components or objects. This results in a blueprint for an initial prototyping, which then undergoes testing and is transferred to the manufacturing of the product [30]. The methodology is well-known in mechanical engineering. It is seldom applied in bioengineering, e.g., for bioprocesses, biosensors, and organ chip device design. In the following, the general workflow in conceptual design when developing a MBR prototype is described.

### 2.1. Design Objectives, User Needs and Specification of Target Values for Design

For achieving success with the conceptual design, a stringent and covering description of the design objective, or the design mission, is the starting point in the process of finding the appropriate design solution. The description of the objective constrains the design. Indirectly, it also encircles existing or potential users [49]. Once the users are known, they can be interrogated about their actual needs and requirements on the design solution [50]. Generally, the user needs for MBRs and OoCs vary with the purpose of the targeted product, which results in different priorities as shown in Table 1. The table elucidates the wide variation in character of needs and requirements that exist, in which some significantly impact the design, while others do not. For example, a certain number of cells are necessary for recapitulating an in vivo function, which sets the minimal size of the MBR-unit; critical biomarker molecules (or protein product, side-products) are produced in amounts possible to measure, which sets another minimal limit for the number of cells required in the MBR-unit; distribution of oxygen and uptake of oxygen in the unit may also be requirements that constrain the design.

Other more specified user needs for an MBR related to the design objective could, for example, involve longevity of use of the MBR, generation of gradients in pO_2_ and nutrients in the device, transformation rates of the cell culture, scalability of units, and flow-through rates. Such needs are highlighted by other authors in this special issue, e.g., by Wrzesinski & Fey [24] on oxygen supply to liver cells in an MBR, by Freyer et al. [23] on 3D microstructure of liver cell MBRs, and by Fernandez et al. [37] on oxygen measurement in MBRs.

Once needs are identified and clearly described, they are also specified with target values or range of values. The target metrics may vary considerably. Table 2 gives examples for a liver-on-a-chip [47]. Note that specified values can be either quantitative or qualitative.

### 2.2. Mapping of the Functional Systems of the Transformation Process and Assessing Their Interactions

**Figure 3:**
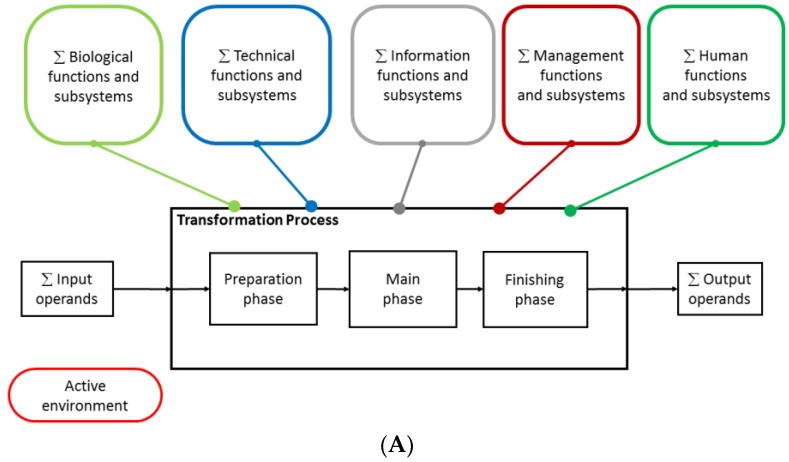

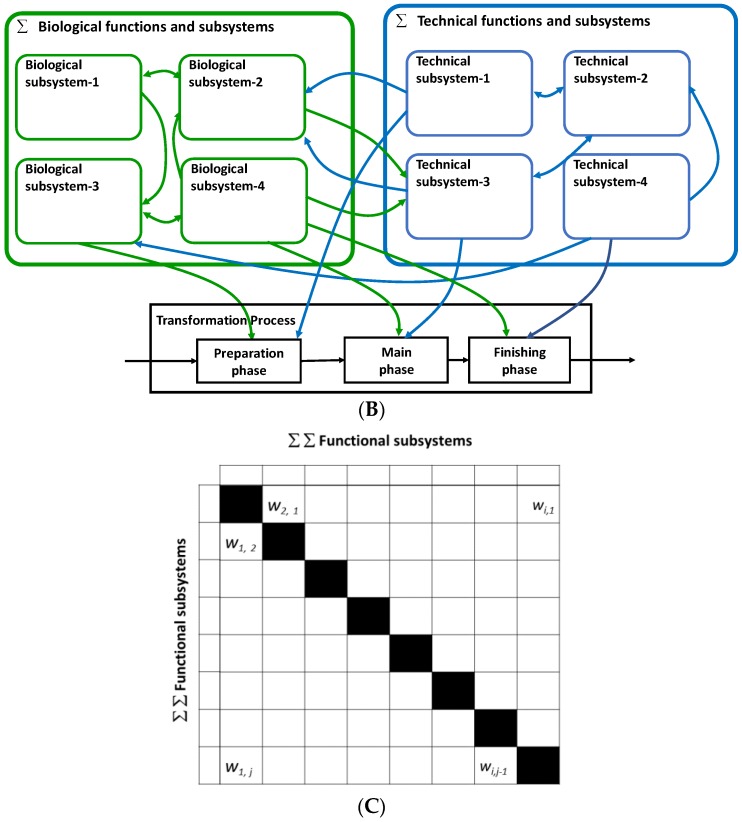
(**A**) Overall Hubka-Eder map showing the transformation process and functional systems; (**B**) a zoom-in of the biological and technical subsystems and their interactions in between and with the transformation process phases; and (**C**) the interaction matrix with assessed interaction effects between subsystems.

A key activity in conceptual design is to describe and establish the structure of the transformation that the designed prototype should perform and what functions are required to perform the transformation. This is done in a graphical representation of the transformation and functions (Figure 3A), a so-called Hubka-Eder map (named after Vladimir Hubka (1924–2006) and Wolfgang Ernst Eder (1930–2017), the originators of this representation) [38]. The essential purpose of this map is (1) to define the transformation process, usually in the phases for preparation, main transformation, and finishing, that should take place in the designed device; and (2) to define the functions required for carrying out the transformation process [48]. The functions are structured into groups of systems, and these are further broken down into functional subsystems (Figure 3B). In a mathematical formalism, this can be described as

$$R=\sum_{i=0}^{n}\times (\sum_{j=0}^{n}FSi,j)$$

in which *FSi*,*j* are the functional sub-systems. The *i* index refers to main functional systems, which we divide into the biological systems, the technical systems, the information systems, the management systems, and the human systems necessary for carrying out the transformation process. To these systems, we also add the unknown surrounding environment, referred to as the active environment, which can influence the functions in ways we are not able to foresee. That could be biological variation, unpredictable sample background, or even influences associated with laws and regulations.

The functional systems and sub-systems interact with the transformation process to drive it forward but can also interact with adjacent functional sub-systems. Understanding the effects of these interactions on the transformation process is the core for accomplishing a functional prototype and is fundamental for making important design decisions. Figure 3C shows a convenient way to represent these interaction effects by ordering them into an interaction matrix, *IM*:

$$IM=\left[\begin{array}{ccc} w1,1\;FS1,1 & \cdots & w1,j\;FS1,j\\ \vdots & \ddots & \vdots \\ wi,1\;FSi,1 & \cdots & wi,j\;FSi,j \end{array}\right]$$

in which the functional sub-systems are represented by a position in the matrix. The weights *wi,j* are values estimating the interaction strengths. These strengths are not precise measures and should only be seen as relative estimates for comparing each *FSi,j*.

When designing MBR devices, technical data from literature can often be helpful. The wealth of published data should, however, be carefully used and valued.

### 2.3. Key Functional Components

**Figure 4:**
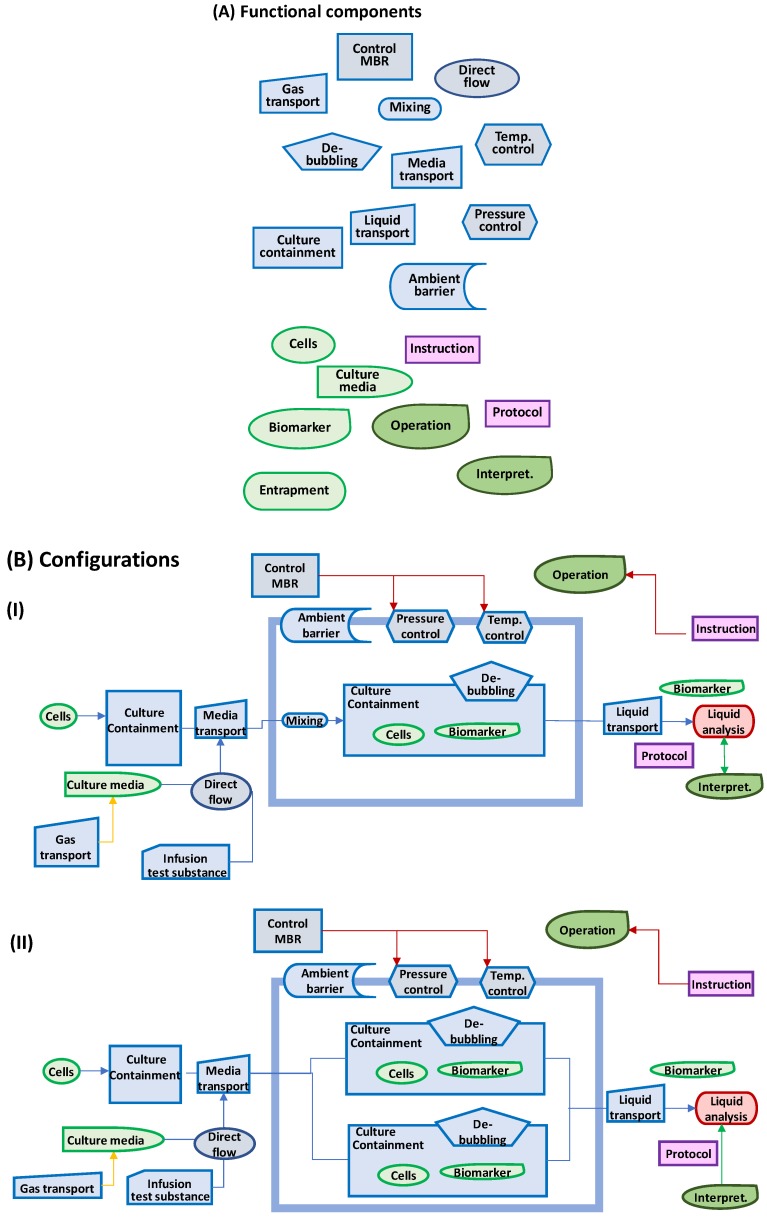
(**A**) Biological (green), technical (blue), and information (red) functional components required in an MBR device as identified from the functional subsystems in the HE-map in Figure 3; (**B**) Two examples of configurations from these functional components in which two MBR units are included in the prototype design.

The Hubka-Eder mapping and the interaction analysis facilitate identification of essential functional components necessary for the design of the prototype [38]. Figure 4A shows a collection of 17 functional components suitable for the design of an MBR prototype, with the functional components grouped in the function systems from the HE-map. Note that a functional component is solely a conceptual object capable of carrying out the function, not a defined physical component, such as a valve or a pump. For example, a fluidic transporter only tells what you want the component to do, not if it is produced by pumping, using a syringe, or utilizing gravitational force. These “neutral”, non-physical, or non-real components allow one to investigate how the functions can be configured. Figure 4B shows two examples of configurations from the 17 functional components. In one configuration (I), cells are transferred to a temperature-controlled space in which the biological systems are preserved in several separately contained and temperature-controlled units with sensor functions. In the other configuration (II), all units are placed in the same temperature-controlled unit, and sensors are shared between the contained cell units. Theoretically, the 17 functional components in Figure 4 can be combined in a multitude of configurations, most of them unrealistic, but a few are realistic and worth investigating further.

### 2.4. Assessment of Configuration Alternatives

The configuration alternatives generated from the functional components should now be compared and assessed versus the user specification target values in Table 2 [40]. Initially, a relatively high number of configuration alternatives can be assessed by a rough screening (20–30 configurations depending on number of components). By that, the number of configurations can be reduced to less than 10, and these can be assessed more thoroughly. Typically, the configuration alternatives can be limited to five or six. We rank their assumed effect on the target specification value; either this is quantitative or qualitative. The ranking is at this stage relative, but could be quantified more exactly, e.g., variability, standard deviation, limit of detection, or analysis time. This will require experimental evidence, e.g., measurements in a test bench in a pre-prototype. Table 3 shows an example of estimated ranking scores for four configuration alternatives. A four-level ranking as shown in the example is sufficient to discriminate the configurations versus the specification values. In Table 3, alternative 2 gets the highest score and is chosen for further development towards a prototype. In the table it is also possible to introduce weight factors to tune the importance of each need in relation to the design objective. For example, low price may have much more impact than size. The balance of the weights is decisive for the final ranking score. A design team must consequently be aware of this and be cautious of how they treat values. However, if they do this, it will become an efficient means with which to perform sensitivity and risk analysis at this early stage of the design.

Once the functionally most feasible design alternative has been selected from the scoring of the user needs, the real physical components replace the functional components [48]. These real components are identified and chosen in a similar selection process. The real component alternatives are screened and scored versus the user needs. Thus, the columns in Table 3 represent real component alternatives that are compared versus the listed user needs and scored.

The real components can be collected from commercial vendors or be specially designed by the team. Of course, commercial components are preferred when they are available and specifications are known. The screening is done similarly and results in a preferred alternative. This alternative will then be prototyped and tested.

## 3. A Micro-Bioreactor for Process Development of Monoclonal Antibody Production

The design mission in this application is to develop an MBR prototype that can be used as a tool in process development of large-scale bio-production of monoclonal antibodies [17,51,52,53,54]. One of the dominating products on today’s biotechnology market is recombinant monoclonal antibodies for use as bio-therapeutics and for diagnostics. The most commonly used production organism is Chinese Hamster Ovary (CHO) cells, but also other cells are used, such as hybridoma and HEK cells [55,56]. Before scaling-up to production, the culture conditions are thoroughly investigated in process development on a small scale [57]. This ensures that the production process is optimized with regards to cell growth rate, antibody production rate, and culture media composition; has optimal values for physiochemical parameters such as temperature, pH, and dissolved oxygen tension in the reactor; is optimal with regard to initiation and propagation of the recombinant expression system in the CHO-cell culture; and applies feeding profiles of nutrients and other growth factors to the bioreactor [58]. In the case of monoclonal antibody production, great concern is devoted to reducing formation of variants of the IgG molecule, such as multimeric and fragmented forms or various glycosylated forms of IgGs [59]. For other bioprocesses, with other expressed proteins, similar modifications are of concern, such as oxygenated, aminated, clipped, or degraded product molecules. All these process development issues are very time-consuming tasks, but are absolutely necessary to scrutinize in the early process R&D.

The MBRs are excellent tools for such R&D work due to their small size and possibility for parallel testing. By using MBRs process, development can be accelerated significantly [11]. Examples of successful steps taken in this direction are emerging mini- or microscale bioreactors with online sensors for measurement of critical process parameters [19,20]. These scaled-downed bioreactor systems have volumes in the range 1–20 mL. Commercial systems are already on the market for suspension cell cultures with a variety of designs [24] (see also Figure 1C).

Conceptual design methodology can efficiently support the design of new MBRs for these purposes [43]. Bioprocess developers have good practical notions of the needs and requirements of MBRs for process development purposes. The most common requirements and specifications for efficient scale-downed optimization work are shown in Table 4.

The table addresses the wide range of cell density or cell number required. It is critical in an MBR to have a volume that is large enough for generating statistically trustworthy data, achieving homogenous fluid in the miniaturized bioreactor system and creating gradients comparable to the scaled-up system. This demand complicates the design for transfer of gaseous molecules, in particular, for oxygen transfer from the gas bubble phase to the liquid phase at the scale of the MBR. Previous designs have either been designed as microtiter-like MBR arrays with sensors in the bottom of each bioreactor-well (e.g., Sartorius, m2p-labs) [19,20] or as separate MBR units with impellers and submerged micro-sensor probes and external pumps (Ambr) [19]. Comparisons with laboratory scale bioreactors of 2–5 L show good correlations, thus indicting that homogeneity of the liquid in the MBR and laboratory scale reactors at this scale is the same when testing mammalian cells, which are the easier cases due their low transfer rate [60]. The table addresses conditions related to these rheological functions of the MBR, as efficiency of mixing of the fluid, transfer of oxygen, addition and withdrawal of nutrients, and sterility demands. These requirements coincide with large-scale cell culture reactors and have also previously been discussed [43].

Furthermore, the cell growth and protein expression in the cell culture in the MBR need to be monitored to provide information about the culture for optimizing conditions and procedures to be transferred to the large-scale process [61,62]. This includes online monitoring of temperature, pH, and dissolved oxygen and offline measurements of components in the bioreactor media, such as product forms, excreted metabolites, and residual nutrients. Finally, the requirements on the MBR design should address management protocols for data analysis and operation, as well as specifications for MBR fabrication (materials, cost, and maintenance needs).

**Figure 5:**
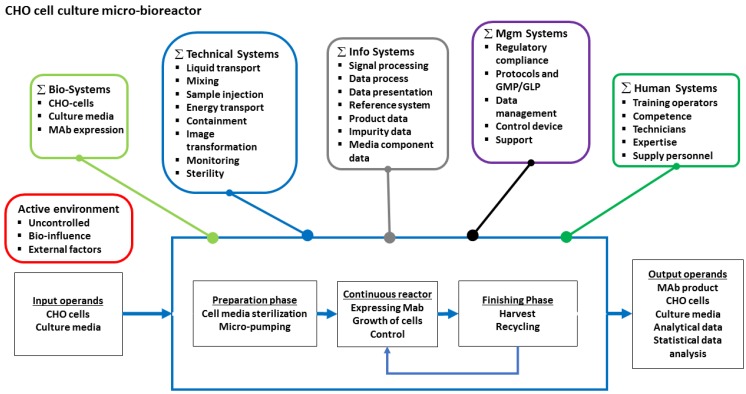
Hubka-Eder map showing the functional systems and subsystems in a continuous recycled MBR for bioprocess optimization of a CHO cell culture producing monoclonal antibody.

The specification of the user requirements in Table 4 suggests a Hubka-Eder map with function systems as depicted in Figure 5. The biological system functions follow from the design mission where the cell line (CHO-cells) has the prime function to express a high titer of the target product (monoclonal IgG) using a recombinant gene construct. The choice of a suitable CHO cell line and expression system is decisive for reaching the production goal. The composition of the culture medium is decisive for the performance of the culture in respect to growth rate, as well as product formation rate. Thus, the interactions between these subsystems, the cell line, the expression system, and the medium are critical issues for the design of the MBR.

The technical system functions are the following: (1) to contain the miniaturized cell culture volume; (2) to provide a sterility barrier towards the environment; (3) to aerate the culture with oxygen a way that is comparable with the large-scale application; (4) to transport culture media with nutrients and excreted product(s) to and from the contained culture; and (5) to measure in real-time the physical and chemical conditions of the culture and its media [57].

The functions of the information system are to collect information from real-time measurements and offline analysis performed in the effluent from the MBR and display these data for process optimization. The information system should also provide information about the performance of the MBR itself and its control functions for temperature, pH and pO_2_, and transport systems (e.g., mixing and pumping).

The functions of the management systems are to provide protocols for MBR operation, computation methods for data analysis, and control of MBR operations through its technical systems. The human systems involved are the laboratory technicians that operates the MBR, the process development engineers and other experts that compute and interpret data from MBR experiments, and service and support personnel that maintain the MBR. These humans interact with all of the other systems and must be able to do that efficiently.

Also shown in the HE-map in Figure 5 is the active environment. This could, for example, be unanticipated biological variations of the performance of the cell line and expression system, partly ascribed to our lack of biological understanding of the complexity of the cell machinery of the CHO cells. Such influences or other influences from the surrounding environment of the MBR should be compensated for, especially by the technical subsystems based on data from the information systems.

All of the systems in the HE-map interact with each other and with the phases of the transformation process. The strengths of these interactions should be analyzed in the interaction matrix (cf., Figure 3C), and those interactions that have high impact should be singled out.

**Figure 6:**
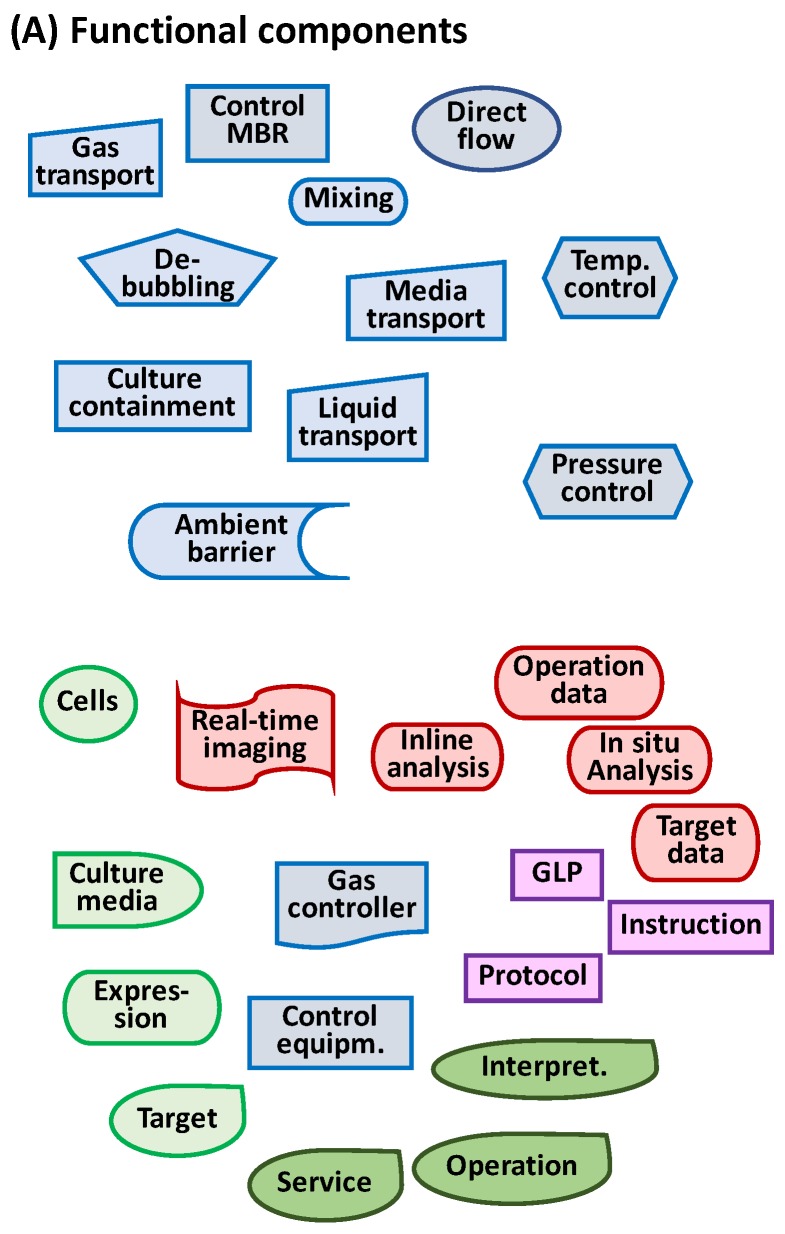

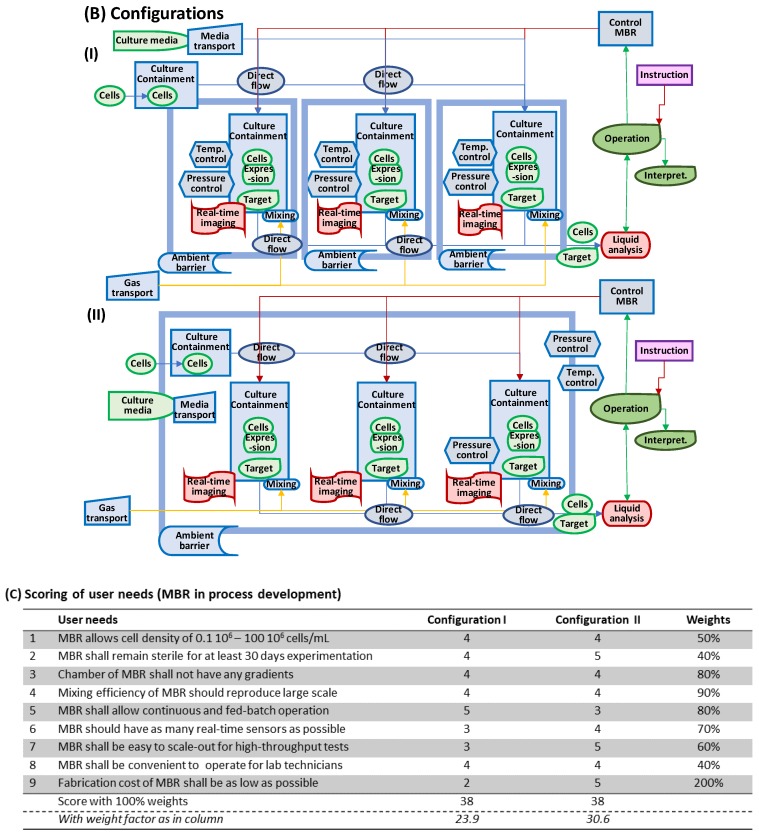
(**A**) Functional components for conceptual design of a MBR for bioprocess development; (**B**) Two configurations of these functional components; (**C**) Comparison and ranking of these two configurations versus user needs in Table 4 in which scores are calculated for weight factors all 100% or as in last column in the table.

Based on this analysis, 16 functional components can be identified that need to be available when configuring the design. Figure 6A shows the functional components for containment, mixing, infusion of media, real-time data sampling, and transportation. The components of the biological functions are limited to the function of the cell line to express IgG, the gene function components, and the functions of the culture medium to enhance growth and IgG production. Information components are the information *per se* and the devices that can generate the information. The management components are software and instructions and rules to be followed. The humans are the individuals able to carry out the functions. These 16 components can easily be arranged in more than ten plausible and realistic configurations. Figure 6B shows two of these configurations, both close to existing MBR products [19,20].

In a full design analysis at least up to 20 configurations would be arranged from the functional components. In configuration I in Figure 6B, each of the parallel MBR units is controlled separately with its own sensors and control units, although they are supervised with one management system. In configuration II, the MBR units are placed in one sensor and control unit. Importantly, the configuration diagram shows only functions for which several physical objects or devices may effectuate the functions.

Comparing these two configurations with the specification list in Table 4 results in a ranking that does not favor any of the configurations (Figure 6C). In the ranking, the weight factors for each specified need have been the same (*w* = 1.0). This may, however, not reflect the actual importance of each need. For example, if the cost is upgraded to *w* = 2, which is a sound assumption, and the other need attributes are tuned down, the total score ranks makes the second configuration (II) alternative the preferred design solution.

**Figure 7:**
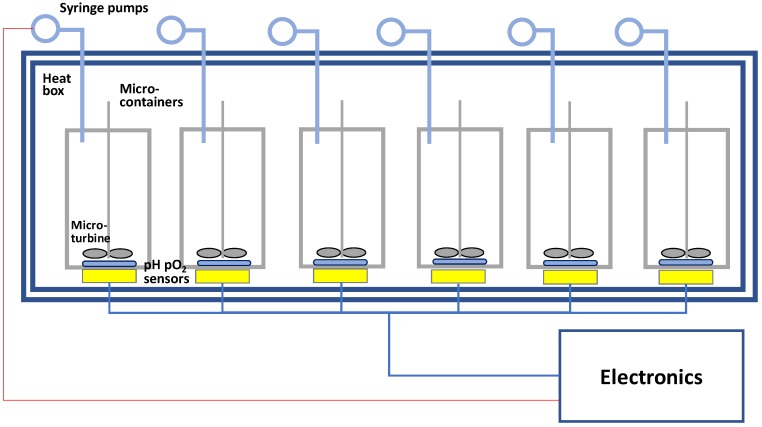
Blueprint of a prototype based on the ranking of components towards the need specifications. An MBR setup with six independent reactors for culture of CHO cells in 2–5 mL scale contained in a thermostat box. Sensors for pH and pO_2_ measure online in each MBR.

In the next step of the design, the chosen conceptual design alternative is translated into a configuration with real physical components replacing the functional components. Thus, common components, such as plastic containers, micro-pumps, temperature and pH sensors, impellers, or robotic racks are introduced and compared. In a second ranking table, the need specifications are used again and component alternatives scored (pumps, gravity mixers, osmotic pumps, and optical and electrical sensor devices) according to their ability to meet the requirements of the specifications (see ranking Table S1 in Supplementary Information) [63,64,65,66,67]. With these ranked components, a first prototype is blueprinted, constructed, and tested (Figure 7). This prototype undergoes testing procedures as commonly done in all product development in the manufacturing industry (cf. detailed description in e.g., Ulrich & Eppinger [40]).

## 4. A Heart-on-a-Chip Micro-Bioreactor for Assessment of Drug Safety and Efficacy

Several designs of heart-on-a-chip (HoC) devices for assessment of clinical in vitro heart models [68,69,70] and for testing of toxicity and efficacy effects of drug substances on cardiac cells have been presented [71,72,73,74,75,76,77]. These studies have shown that the HoCs devices have the potential to facilitate and shorten drug development in the pharmaceutical industry when the HoCs can accurately reflect relevant in vivo conditions in human heart tissues related to critical drug effects. The challenge is to accomplish theses in vivo-line conditions in the HoCs. In this respect, the access to induced pluripotent stem cells (iPSC) from stratified patient groups with different genetic background makes iPSCs an attractive approach that bypasses animal testing with lesser relevance [78]. These prerequisites and ambitions have guided most recent efforts in designing HoC devices. Examples are studies with electrical or visual recording of the heart’s beating rate with cardiac bodies [78] exposed to drug substances at varying concentrations [79].

In the example shown here, the design objective is to design a HoC prototype using commercially available, iPSC-derived cardiomyocytes [80] or cardiomyocytes developed in their own lab, e.g., at a research institute or a R&D unit at a drug company [81]. The purpose of the design of the HoC device is to assess effects of drug candidates, either for cardiac chemotherapy or other therapies in which the heart is affected by the drug.

The cardiomyocytes in the HoC prototype will experience the same spatiotemporal conditions as in the in vivo heart tissues. This requires a 3-dimensional cellular microstructure inside the HoC flow-through chamber, in which other cells in the cardiac tissue shall be included as well. This could either be assemblies of co-cultured cells or assembled scaffold structure in materials such as hydrogels or other biopolymers. The perfusion around the in vitro cell assembly shall mimic the conditions in the in vivo heart tissue, which requires that the microfluidic conditions in the HoC are precisely designed and dimensioned.

Table 5 lists commonly expressed user needs related to drug testing on the design in line with the cellular prerequisites highlighted above, together with several others for technical, analytical, and management functions. Also, a few business-related needs are mentioned, although the conceptual design is not at this stage aiming at a market analysis of a HoC; focus is on utility and technical performance. However, manufacturing and marketing must inevitable sooner or later be addressed in the product development process, in which the design alternatives may play a decisive role in manufacturing, marketing, and price.

Clearly, there are many similarities between the user needs for a HoC device shown in Table 5 and the previous MBR design for process development (Table 4). However, the requirements on the biological systems are in the HoC more demanding due to the complex tissue architecture of the cardiac tissue. The minimal tissue equivalent, i.e., the smallest number of cells that can recapitulate the active in vivo heart tissue unit, which must be defined and realized in the HoC chambers, is a key issue in the prototype design. Also, supportive cells, e.g., fibroblasts and nerve cells for synchronized contraction (Purkinje cells), and the structure of the myocardial extracellular matrix (ECM) should be a part of that equivalent. The interaction with the culture media plays, in this respect, a critical role in maintaining the cells’ responses. The technical system functions as described in the previous MBR example remain largely unchanged for a HoC prototype, but are required to be extended with electrical or optical measurement functions for recording the contractions of the cell clusters. The information functions should, in the HoC, include methods to analyze the motility of the cells or cell clusters.

**Figure 8:**
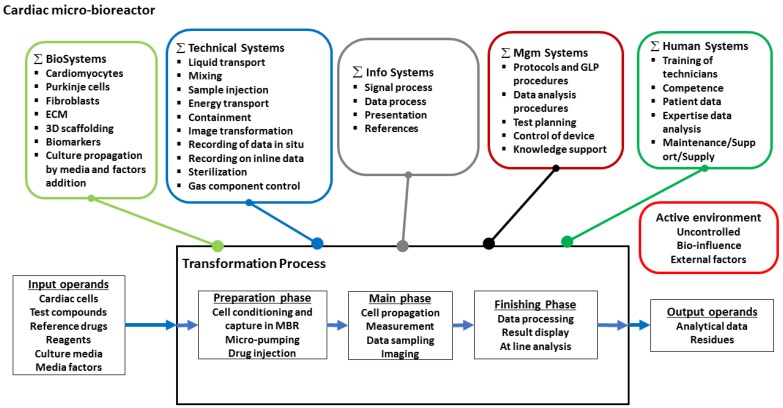
Hubka-Eder map showing the functional systems and subsystems that could be involved in a heart-on-a-chip MBR for purposes indicated in Table 5.

These user requirements suggest a Hubka-Eder map with function systems as depicted in Figure 8. The biological system functions (∑ BioSystems) have additional subsystems for building up the heart tissue units. The other ∑ Systems remain much the same as in the previous example. The interactions between the ∑ BioSystems and the other systems will, in this application, show a higher degree of complexity due to the three cell types and the myocardial ECM. The systems’ interactions with the three phases of the transformation process are in some parts similar, in others different. The preparation phase has a key role in establishing the tissue structure to generate contraction between cardiomyocytes. The sensors for monitoring the cell aggregates in situ are critical for generating the information that the computational procedures in the management system shall process further into interpretable data.

**Figure 9:**
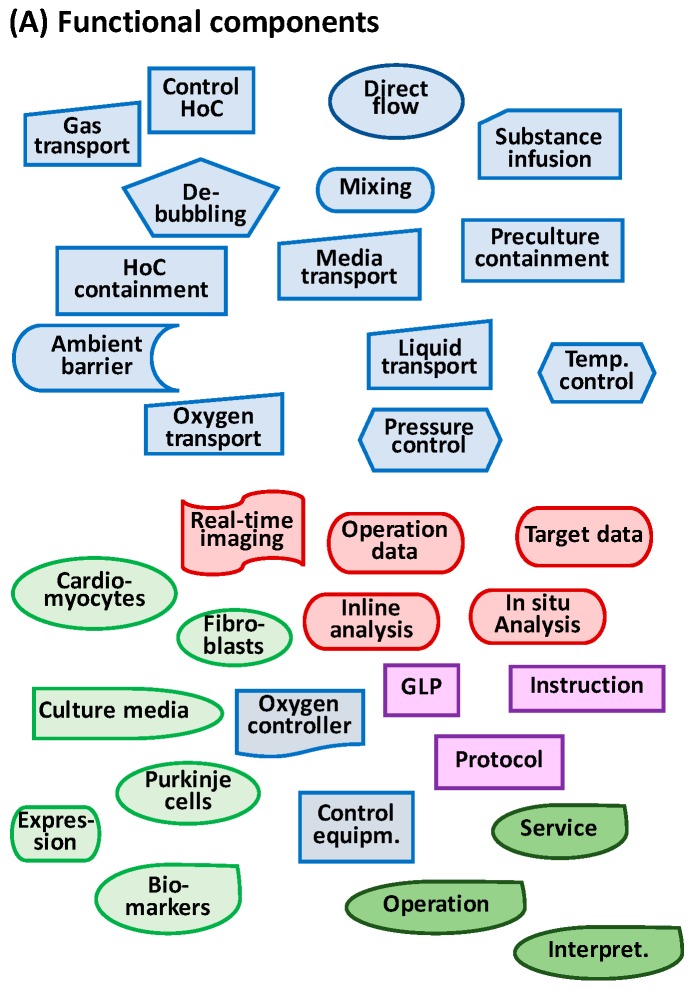

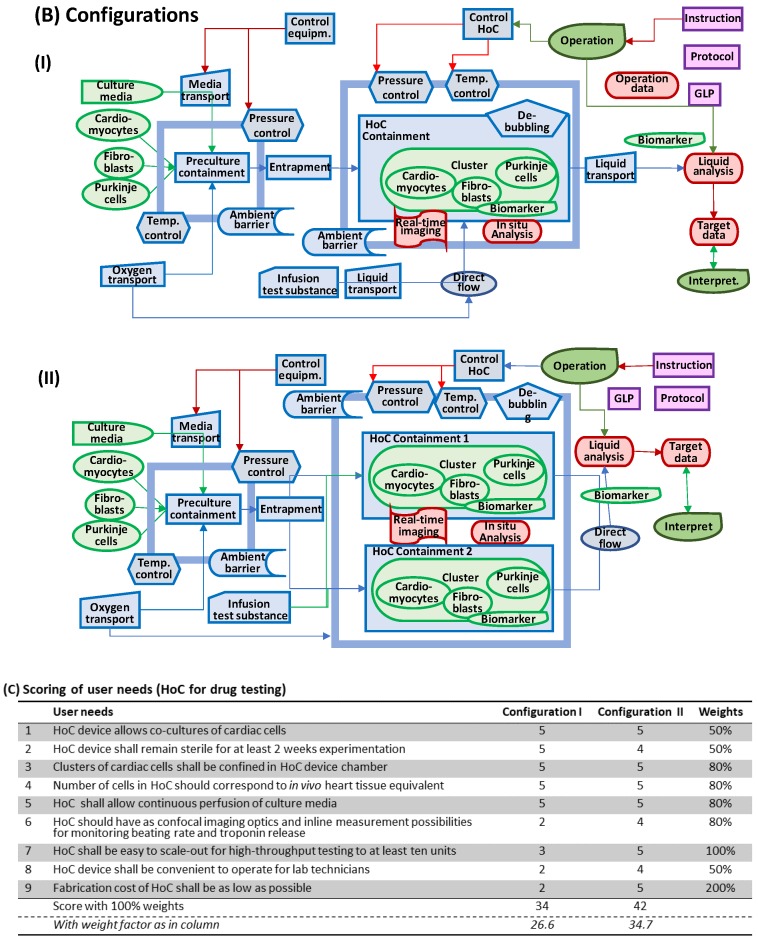
(**A**) Functional components required for a design of a MBR with cardiac cells based on the functional subsystems in Figure 7. (**B**) Two possible configurations of these functional components. (**C**) A comparison of the two configurations with the user needs in Table 4 in which scores are calculated for weight factors (all of which are 100%) or as shown in last column in the table.

From the HE-map, the functional components related to the biological subsystems, i.e., cells and tissue functions, are identified (Figure 9A). These components can be arranged in a multitude of configurations (Figure 6B shows two possibilities of configurations). The upper configuration (I) is a design in which all cell types are confined in one body and these bodies are contained in the chip. The lower configuration (II) is a design based on parallel containment units in which cardiac bodies are kept apart. Additional configurations are, in this HoC application, more motivated to generate due to the extended complexity of the biological systems (not shown here) When comparing the two configurations in Figure 6 with the specification results in a ranking (with *w* = 1.0), which favors Configuration II when using the same weights for all need attributes (Figure 6C). Interestingly, if the weights are differentiated according to the importance of the need attributes, the ranking order remains.

**Figure 10:**
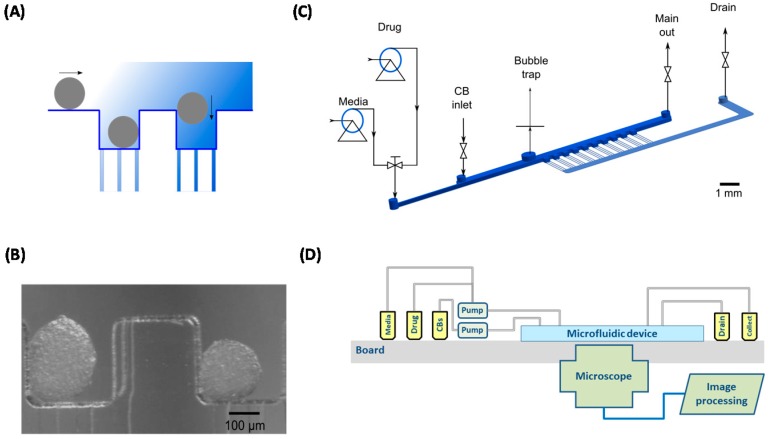
A blueprint of a Heart-on-Chip device designed based on the HE-map conceptual analysis in Figure 8 and Figure 9 and the scoring of components in Table S2 (from Bergström et al. [79]). (**A**) Micro-wells with cardiac bodies; (**B**) micrograph showing two beating cardiac bodies in two wells; (**C**) graphic of the microfluidic device, and (**D**) the HoC setup.

Next, the chosen conceptual design alternative (Configuration II) is translated into a blueprint of a heart-on-a-chip prototype in which a selection of real physical components is configured. Table S2 (Supplementary Materials) [82,83,84,85,86,87] shows the ranking of real components/devices and how these components are compared and scored versus the same need attributes that originally were identified by the users. The total scoring guides the team in the configuration of the HoC prototype using the components with the highest scores. Figure 10 shows an example of a blueprint of a previously published HoC device [79] with components fabricated in PDMS material using soft lithography, with a syringe pump, with an in situ microscopy, and with software analyzing the beating pattern of the cardiac cell clusters. The device is based on the guidance from the conceptual methodology. An alternative HoC design using commercial plastic components is described elsewhere in this issue [84].

## 5. Conclusions and Outlook

Although the complexity of the designed products varies, the same conceptual design methodology can be applied when designing MBRs and OoCs. The two MBR examples discussed in this review—(1) an MBR prototype for process development of recombinant mammalian cells for monoclonal antibody production and (2) an HoC prototype for drug testing of cardiac cells—elucidate this at two levels of complexity of biological systems.

Other OoC devices with higher degrees of complexity and multi-compartmental organization, such as brain tissues (e.g., blood-brain-barrier (BBB) chips) and the human eye (eye-on-a-chip) [88,89], exhibit design challenges beyond what is shown here for the HoC. For example, to recapitulate the membrane structure of blood capillaries in the brain or to recapitulate the functions of the multi-layer cellular assemblies of the cornea [29] and retina [90] or the blinking function of the eyelid [91] requires elaborate design efforts. Especially, the integration with the surrounding biopolymers (ECMs and membranes) forming scaffolds for realizing the 3D architecture of the organ then becomes an essential function in the design. Design work with the BBB- and eye-on-a-chip devices could benefit from the structural approach of conceptual design methodology.

One of the most important issues in the MBR design is to successfully mimic reality. For the heart-on-a-chip case, as well as other OoCs, that is about mimicking conditions in vivo of a human tissue. For the microbioreactor with a recombinant mammalian cell culture producing monoclonal antibodies, it is to mimic the conditions of a large-scale bioreactor with the same culture in the MBR. The ability of the MBR prototype to do this must be assessed experimentally by comparing data derived from the MBRs with data from the real system, i.e., the in vivo heart tissue or the large-scale bioreactor. Only when these data coincide sufficiently well, the prototype design can be considered successful. The bioanalytical possibilities to verify the correlation between the real system and the MBRs are considerably more demanding for the in vivo systems, e.g., the human heart tissue in a patient, and for some parameters this is not even possible in practice. For the large-scale bioreactor this is comparatively easy. Thus, the outcome of the design of a HoC prototype may not be satisfactorily assessed. This will most probably be the case for the mammalian cell culture MBR.

The two design examples in this review exhibit modest cellular complexity. This is the case especially for the MBR with the recombinant mammalian cells, which is a homogeneous mono-cellular culture; the cardiac chip is more complex, as it has a multi-cellular structure, but is limited to three cell types (cardiomyocytes, fibroblasts, and nerve cells). Still, the network of interactions between functions and real components in the design becomes significant and demanding to comprehend for the design team. This justifies the conceptual methodology with its systematic evaluation of design alternatives. The conceptual approach contributes to reducing unsuccessful prototyping of devices and facilitates the finding of favorable designs for design purposes.

Other supporting methods, such as mathematical modeling, should, however, not be neglected as useful tools to support the conceptual design. Mathematical models and dynamic simulations can be very useful, e.g., for estimating rates and dimensions in the cellular system of the organ [92]. Also, computer-aided design combined with conceptual design has proved useful in bioengineering, for example, with stem cell-derived cardiomyocytes [93]. Thus, other established engineering design methods should therefore support and complement conceptual design methods, in parallel or initiated by the conceptual directions.

## Figures and Tables

**Table 1:** Examples of common user needs of micro-bioreactors and organ-on-chips.

Users’ Needs and Requirements	Rationales or Examples	Priority
Maintain human organ cells with number of a corresponding human organ equivalents	Lower number of cells in a reactor unit would not show relevant data	10
Cells shall maintain the same functionality in vitro as they do in vivo	In most assays, the functionality is the target end-point to be observed	10
A multi-cellular system should be recapitulated in the MBR/OoC	A human organ or tissue is in vivo, like if it interacts with adjacent cells	9
Excreted metabolites shall be analysed in situ or at line with sensitive analytical means	Amounts of analytes produced in the MBR are minutes due to scale	9
Cell densities equivalent to an industrial production system	In-process development should have cell concentrations on a large scale	8
Material properties of MBR should not interfere with the biological transformation	Some materials are toxic, absorb drugs, or affect gradients of gases	8
Microfluidic conditions in the device should not harm the cells capacity	Shear force in the micro-reactor shall not change cells functional behaviour	7
The MBR/OoC shall be operable with stable performance over extended time periods	Short-term acute effects (<1 day) are of lesser value than chronic (>14 days)	7
Compounds should be exposed to cells or cell organoids in a relevant way	Diffusion, shear, and gradients in MBR should reproduce in vivo perfusion	6
Allow controlled addition of media factors	The exposure of factors	2
Gradients in the MBR of O_2_, CO_2_, pressure, and temperature should be in vivo-like	Variations in gradients are known to influence cellular response	1

**Table 2:** User needs of a micro-bioreactor with organ cells.

User Needs	Target Metrics (Examples)	Specification (Examples)
MBR shall have multi-cellular systems	At least three cell types	Hepatocytes, Kupfer cells, fibroblast
Cells shall have in vivo-like functionality	Cells per MBR unit	50,000–75,000 cells
Cells as in human organ equivalent	Cells in equivalent	25,000 cells
Extracellular matrix	Hydrogel type	Matrigel or RGD-PEG
Flow of nutrients	Shear force number	
Measurement be extended time periods	Days	>10 days
Controlled addition of growth factors	Pump rates	
Microscope In situ inspection	Microscopic resolution	±10 nm
Sampling of effluent fluid	Number sampling ports	3
Oxygen transfer	Dissolved oxygen tension	More than 10%
Oxygen permeability of device material	mg O_2_ per mL and hour	
Material properties of device	Porosity	25–30%
Recycling of media	Recycling ratio	1–2

**Table 3:** Example of ranking of design alternatives versus target specification metrics ^1^.

Design Alternatives User Needs	Alternative 1	Alternative 2	Alternative 3	Alternative 4
Allow co-culture of cells	••	•••	••	•
Cells have in vivo-like functionality	••	•••	•	•
Number of cells in device as in an in vivo equivalent	•	••	••	•••
Extracellular matrix possible to mimic	•••	•••	•••	•••
Continuous flow of nutrients	••	•••	••	•••
Measurement periods up to 3 weeks	-	•	•••	-
Exposure of test compounds to cells	•••	•••	•••	•••
Allow controlled addition of growth factors	•••	•••	-	••
In situ inspection with confocal microscope without interference	•••	•••	-	-
Sampling of effluent fluid	•••	•••	•••	•••
Oxygen transfer through device	•	•	•••	•••
Liquid permeability of device	••	••	••	-
Device shall allow recycling of media or exposed compounds	•••	•••	•••	•••
Material properties of device not interfering	•	•	•	••
Recycling of outlet flow	•••	•••	-	•
Total score of ranking	32	37	28	28

^1^ Compliance with user needs ••• high; •• partly possible; • low or uncertain; and - none or impossible.

**Table 4:** User needs of a micro-bioreactor for process development of mammalian cell cultures.

User Needs	Target Metrics	Specification
Biological functions		
Mammalian cells shall be used	Cell type	CHO, HEK cells
Concentration range of cell culture	Cell/mL	10,000–10,000,000
Expression of extracellular protein	Proteins expressed	IgGs
Same culture media shall promote both growth and expression	Type of media to be used	Serum-free medium
Culture time	Days	7–14 days
Technical functions		
Gentle well-distributed mixing	Shaken or stirred	Shaken
In situ inspection with confocal microscope without interference	Yes/No	Yes
Sampling of effluent fluid	Offline/inline	Offline
Oxygen transfer	k_L_a value for OTR	>100 h^−1^
Permeability of device	Oxygen permeability (%)	<1%
Material properties of device	Surface hydrophobicity (angle)	10 degree
Information functions		
Online information about physical conditions in the MBR	Sensor types	Temp., pH, pO_2_
Offline information about content of culture media	Analytes analyzed offline	All monomers in culture media
Offline information about IgG forms	Analytes analyzed offline	IgG forms
Low fabrication cost	Percentage of the sales price	>10%

**Table 5:** User needs of a HoC micro-bioreactor with cardiac cells intended for compound testing.

User Needs	Target Metrics	Specification
Biological needs		
Co-culture of cardiomyocytes/fibroblasts	Number other cell types than CM	2–4 other cell types
Cardiomyocyte assemblies beating	Beats per minutes (bpm)	30–100
cardiac cells clustered in aggregate	Number of cells per aggregate	500–1000
Sufficient cells in HoC to generate measurable signals	Cardiac cells per HoC chamber	500,000–1,000,000
Extracellular matrix created inside MBR	Type of biomaterials	PEG, Matrigel
Technical needs		
Shear force on cells corresponds in vivo of liquid media (nutrients, test solutions)	Distribution of flow rates in HoC Psi/cm	±10%
Thermostable condition for cells in HoC	Temperature range inside HoC	35–38 °C
Sampling ports for HoC effluent fluid	No. of ports and where	in: 2–3, out: 1
Oxygen transfer to cardiac bodies	Dissolved oxygen tension in aggregates	above 5%
Non-toxic fabrication materials of MBR	Type of materials	Plastics, metal
Sterile conditions	Sterility time	2 weeks
Information needs	Methods; performance	Confocal microscopy Magnification ×50
In situ non-destructive inspection of cells		
In situ observation of biomarkers		
Measurement acquisition online	Methods; performance	HCI
Inline monitoring of excreted substances	Methods; performance	MS, immunosensor
Product and manufacturing requirements		
Production cost per device/10,000 per year	EUR/unit	2–4 EUR
Consumable cost per assay	Range EUR/assay	1–5 EUR
Technician training time	Days	3 days

## Supplementary Materials

The following are available online at http://www.mdpi.com/2306-5354/5/3/56/s1, Table S1: Scoring of user needs versus real components for MBR for process development Configuration I, Table S2: Scoring of user needs versus real components for Heart-on-a-Chip Configuration I.
