# Supplementary material for: Conceptual Design of Micro-Bioreactors and Organ-on-Chips for Studies of Cell Cultures
Source: Bioengineering (Basel). 2018 Jul 19;5(3):56. doi: 10.3390/bioengineering5030056 (PMC6164921; doi:10.3390/bioengineering5030056)
Supplement: Supplementary file 1 [file bioengineering-05-00056-s001.pdf]

Table S1: Scoring of user needs versus real components for MBR for process development Configuration I

Needs	Σ BioS			Σ TS						Σ IS		
	BioS $_{entrapment}$			TS $_{transport}$			TS $_{containment}$			IS $_{online\ data}$		
	Suspen-sion[1]	Porous microcarrier[2]	Hollow-fiber[2]	Peristaltic pump	Syringe Pump[3]	Electrooso-tic pump[4]	Plastic tube array[7]	Steel microwell[7]	Plastic microreactor[7]	ISM[8]	Capa-citance[9]	Online HPLC
MBR allows cell density of 0.1 10^6 – 100 10^6 cells/mL	●●●	●●	●●	●●●	●●	●	●●●	●●●	●●●	●●●	●	●●
MBR shall remain sterile for at least 30 days experimentation	●●●	●	●●	●●●	●●●	●●	●●●	●●●	●●●	●●	●●	●●
Chamber of MBR shall not have any gradients	●●●	●●	●	●●●	●●●	●●	●	●●	●●●	●●●	●	●●●
Mixing efficiency of MBR should reproduce large scale	●●●	●	●	●●●	●●●	●●●	●	●●	●●●	●●	●●	●●●
MBR shall allow continuous and fed-batch operation	●●●	●●●	●	●●●	●●●	●	●	●	●●●	●●●	●●●	●●●
MBR should have as many real-time sensors as possible	●●●	●	●	●●●	●●●	●●●	●	●	●●	●●	●	●
MBR shall be easy to scale-out for high-throughput tests	●●●	●●	●●●	●●●	●●●	●●●	●●●	●●	●●	●	●	●
MBR shall be convenient to operate for lab technicians	●●●	●	●	●●●	●●●	●●●	●●●	●●●	●●●	●●●	●●	●●
Fabrication cost of MBR shall be as low as possible	●●●	●	●	●	●	●●●	●●●	●	●●	●	●●	●
TOTAL SCORE	27	14	13	25	24	20	19	18	24	20	15	20

Comments to table: [1] Suspension culture usually preferred [63]. [2] Common alternatives are microcarrier cultures and hollow-fibers [63]. [3]Syringe pump for precision pumping [64]. [4]Electroosmotic pump delivering an oscillating flow [65]. [7] Examples of MBR vessels are found in current commercial devices [19,20]. [8] In situ microscopy for real-time monitoring [66]. [9]For example, dielectric probes [67].

Table S2: Scoring of user needs versus real components for Heart-on-a-Chip Configuration I

	Σ BioS			Σ TS						Σ IS		
	BioS entrapment			TS transport			TS containment			IS beating rate		
**Needs**	Cardiac Bodies[1]	3D-membrane	3D-matrigel	Gravity transport[2]	Syringe Injection[3]	Electroosotic pump [4]	PDMS 10-well array[5]	PE 96-microwell[6]	PDMS Microcolumn[7]	ISM[8]	HCS[9]	MEA[10]
HoC device allows co-cultures of cardiac cells	●●●	●●	●●	●●●	●●●	●●	●●●	●●	●●	●●●	●●●	●
HoC device shall remain sterile for > 2 weeks experimentation	●●	●●	●●	●●●	●	●●●	●●	●●	●●	●●●	●●●	●
Clusters of cardiac cells shall be confined in HoC device chamber	●●●	●	●●	●●●	●	●●●	●●●	●●●	●●●	●●●	●●●	●●●
Cell number in HoC shall equal *in vivo* heart tissue equivalent	●●●	●●	●●	●●●	●●●	●●●	●●●	●	●	●●●	●●●	●●●
HoC shall allow continuous perfusion of culture media	●●●	●●	●	●●●	●	●●●	●●●	●●	●●	●●●	●●●	●●
HoC shall have confocal imaging optics and inline measurement for monitoring beating rate and troponin release	●	●●	●●●	●●●	●●●	●●●	●●●	●	●	-	●●●	-
HoC shall be easy to scale-out for high-throughput testing to at least ten units	●●●	●	●●	●●●	●●●	●	●●	●●●	●●	●	●●●	●●
HoC device shall be convenient to operate for lab technicians	●●●	●●	●●	●●●	●●	●●	●●	●●●	●●	●●●	●●●	●●●
Fabrication cost of HoC shall be as low as possible	●●●	●●	●●	●●●	●●●	●●●	●●●	●●●	●●●	●●●	●	●●
TOTAL SCORE	24	16	18	27	20	23	24	21	19	22	25	17

Comments to table: [1] Co-culture of cell types prepared in spheroids (cardiac bodies) as described in [72]. [2] Rocker used to transport liquid in linear channel [86]. [3]Syringe pump for precision pumping [81]. [4]Electroosmotic pump delivering an oscillating flow [65]. [6]MIMETAS system [88]. [7] PDMS microfluidic channel packed with entrapped cells [87]. [8] In situ microscopy for real-time monitoring [94]. [9]High content screening with e.g. Perkin Elmer OPERA system [89]. [10]Microelectroarray for cardiac cells [84].
